# Isolation of *Acanthamoeba* T5 from Water: Characterization of Its Pathogenic Potential, Including the Production of Extracellular Vesicles

**DOI:** 10.3390/pathogens9020144

**Published:** 2020-02-21

**Authors:** Lissette Retana Moreira, Daniel Vargas Ramírez, Fátima Linares, Alexa Prescilla Ledezma, Annette Vaglio Garro, Antonio Osuna, Jacob Lorenzo Morales, Elizabeth Abrahams Sandí

**Affiliations:** 1 Departamento de Parasitología, Universidad de Costa Rica, San Pedro, Montes de Oca 2060, Costa Rica; danvarram@hotmail.com; 2 Centro de Investigación en Enfermedades Tropicales (CIET), Universidad de Costa Rica, San Pedro, Montes de Oca 2060, Costa Rica; ann04vg@gmail.com; 3 Centro de Instrumentación Científica (CIC), Universidad de Granada, Granada 18071, Spain; flinaor@ugr.es; 4 Departamento de Parasitología, Grupo de Bioquímica y Parasitología Molecular (CTS 183), Instituto de Biotecnología, Campus de Fuentenueva, Universidad de Granada, Granada 18071, Spain; alexa_prescilla@yahoo.es (A.P.L.); aosuna@ugr.es (A.O.); 5 Instituto Universitario de Enfermedades Tropicales y Salud Pública de Canarias, Universidad de La Laguna, Avda. Astrofísico Fco. Sánchez, S/N, La Laguna, Tenerife, Islas Canarias 38203, Spain; jmlorenz@ull.edu.es; 6 Departamento de Obstetricia, Ginecología, Pediatría, Medicina Preventiva y Salud Pública, Toxicología, Medicina Legal y Forense y Parasitología, Universidad de La Laguna, Avda. Astrofísico Fco. Sánchez, S/N, La Laguna, Tenerife, Islas Canarias 38203, Spain

**Keywords:** pathogenic potential, proteases, cytopathic effect, extracellular vesicles, atomic force microscopy

## Abstract

*Acanthamoeba* is a genus of free-living amoebae widely distributed in nature, associated with the development of encephalitis and keratitis. Despite the fact that it is common to find genotype T5 in environmental samples, only a few cases have been associated with clinical cases in humans. The wide distribution of *Acanthamoeba*, the characteristic of being amphizoic and the severity of the disease motivate researchers to focus on the isolation of these organisms, but also in demonstrating direct and indirect factors that could indicate a possible pathogenic potential. Here, we performed the characterization of the pathogenic potential of an *Acanthamoeba* T5 isolate collected from a water source in a hospital. Osmo- and thermotolerance, the secretion of proteases and the effect of trophozoites over cell monolayers were analyzed by different methodologies. Additionally, we confirm the secretion of extracellular vesicles (EVs) of this isolate incubated at two different temperatures, and the presence of serine and cysteine proteases in these vesicles. Finally, using atomic force microscopy, we determined some nanomechanical properties of the secreted vesicles and found a higher value of adhesion in the EVs obtained at 37 °C, which could have implications in the parasite´s survival and damaging potential in two different biological environments.

## 1. Introduction

Of the 22 genotypes of *Acanthamoeba* described to date, T4 and T5 are the most frequently isolated from nature [1]. Clinically, it is known that *Acanthamoeba* produces keratitis and encephalitis. Some cases of pulmonary and cutaneous manifestations have also been reported [2,3]. Genotype T4 is responsible for 90% of these clinical cases, while the rest of them are produced mostly by genotypes T2, T3, T6, T11, T13 and T15 [4,5,6,7,8]. 

Despite the fact that it is very common to find *Acanthamoeba* T5 in environmental samples, it was not until 2006 that this genotype was involved in clinical cases in humans. To date, only three cases of keratitis due to this genotype have been reported [9,10,11], one fatal disseminated Acanthamoebiasis case in a patient previously submitted for a heart transplant [12] and one case of encephalitis in an immunocompetent patient [13]. The reasons for the scarcity of cases related to this genotype are unknown. It is important to highlight that, in the description of these cases, the isolated amoebae were very aggressive [13] or resistant to the treatment usually employed for *Acanthamoeba* keratitis [14]. 

The wide distribution of *Acanthamoeba*, as well as the characteristic of being amphizoic and the severity of the disease that it causes, has motivated researchers to focus not only in the isolation of these organisms, but in demonstrating the presence of direct and indirect factors that could indicate a possible pathogenic potential [15,16,17,18,19]. Most of the literature refers to osmotolerance and thermotolerance, the production of proteases and cytopathic effects in vitro as the main factors related to pathogenicity. Recently, the participation of extracellular vesicles (EVs) in intercellular communication and in the pathogenesis of diverse organisms was proven. 

EVs are a diverse group of nanoparticles released by both prokaryotic and eukaryotic cells, surrounded by a phospholipid bilayer [20] and contain a bioactive cargo of proteins, metabolites, nucleic acids (DNA and RNA) and lipids. Depending on their composition, size and biogenesis, these EVs could be classified in exosomes (< 100 nm); microvesicles, microparticles or ectosomes (100 nm–1 μm) and apoptotic bodies (> 2 μm) [21,22]. Besides intercellular communication and pathogenesis, EVs have been involved in immunomodulation, evasion of the immune response and metastasis, among others. In the case of protozoan parasites, they have been described specially in *Trypanosoma cruzi* [23,24,25], *Leishmania* [26,27], *Toxoplasma gondii* [28,29], *Eimeria tenella* [30], *Cryptosporidium parvum* [31], *Trichomonas vaginalis* [32], *Giardia intestinalis* [33] and recently, in *Acanthamoeba castellanii,* a free-living amoeba [34,35].

The aim of this work was to perform the complete characterization of an *Acanthamoeba* genotype T5 isolated from a water sample collected in the Internal Medicine Unit from a hospital. We focused the analysis on evaluating the presence of virulence factors related to pathogenic potential in *Acanthamoeba*, including the secretion of extracellular vesicles. 

## 2. Results

### 2.1. Isolate Genotyping Reveals a Thermotolerant Acanthamoeba T5 Isolate

The molecular analysis after the amplification of the DF3 region of the 18S rDNA gene determined that this *Acanthamoeba,* isolated from the water of a hospital, belonged to genotype T5 and showed 98% homology with *Acanthamoeba lenticulata*. The amoeba was able to grow at 37 °C but not at 40 °C, and was nonosmotolerant (results not shown). The sequence was deposited into the GenBank with the accession number: MH82415.

### 2.2. Virulence Factors of the Acanthamoeba T5 Isolate

#### 2.2.1. *Acanthamoeba* T5 Secretes Active Serine and Cysteine Proteases 

**Figure 1:**
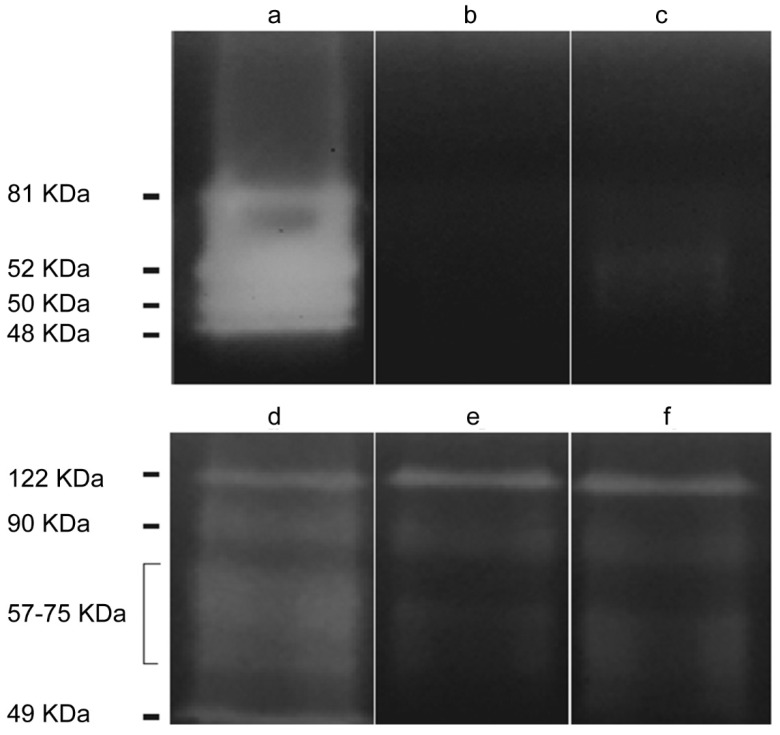
Protease zymogram for the *Acanthamoeba* T5 isolate. Lanes: (**a**) *Acanthamoeba* conditioned medium (ACM), (**b**) ACM incubated with phenylmethylsulfonyl fluoride (PMSF) (inhibitor of serine proteases), (**c**) ACM incubated with 2-iodoacetamide (inhibitor of cysteine proteases), (**d**) crude extract of trophozoites, (**e**) crude extract of trophozoites incubated with PMSF and (**f**) crude extract of trophozoites incubated with 2-iodoacetamide.

The presence of proteases with molecular weights higher than ≈ 45 kDa was observed in the conditioned medium and in the crude extract of trophozoites (Figure 1). The molecular weight of the proteases in the conditioned medium were ≈ 48, ≈ 50, ≈ 52 and ≈ 80 kDa, while proteases of ≈ 48, ≈ 80, ≈ 122 kDa and a wide diffuse area of gelatin digestion between ≈ 57 and ≈ 75 kDa were observed in the crude extract. Both phenylmethylsulfonyl fluoride (PMSF) (inhibitor of serine proteases) and 2-iodoacetamide (inhibitor of cysteine proteases) inhibited protease activity of the conditioned medium almost completely, except in the case of the band of approximately 50–52 kDa that was only partially inhibited. Regarding the crude extract of trophozoites, there was a partial inhibition of protease activity in the ≈ 48 and ≈ 57–80 kDa bands when incubating with each one of these inhibitors. None of the inhibitors acted over the 122 kDa band. Finally, EDTA (inhibitor of metalloproteases) did not produce an inhibition of the protease activity in the samples assayed (results not shown).

#### 2.2.2. Cytopathic Effect of *Acanthamoeba* T5 over MDCK and Vero Cell Lines

**Figure 2:**
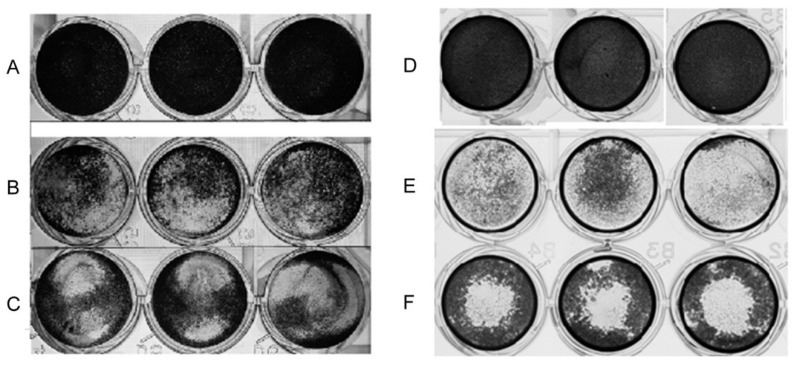
Crystal violet stain that shows the cytopathic effect of *Acanthamoeba* T5 over Madin-Darby canine kidney (MDCK) and Vero cells. Amoebae were incubated with MDCK or Vero cells in 24-well plates for 24 h at 37 °C and their cytopathic effect was observed using the crystal violet stain. Lanes: (**A**) MDCK cell control, (**B**) MDCK cells incubated with *Acanthamoeba* CLC-16 (control strain of cytopathic effect), (**C**) MDCK cells incubated with *Acanthamoeba* T5; (**D**) Vero cell control, (**E**) Vero cells incubated with *Acanthamoeba* Neff (control strain of cytopathic effect) and (**F**) Vero cells incubated with *Acanthamoeba* T5. Images are representative of experiments performed in triplicate.

The cytopathic effect in vitro of the *Acanthamoeba* T5 isolate was determined using the crystal violet and the fluorescent Hoechst 33342 stains. Regarding the crystal violet stain, after 24 h of incubation of the cells with the amoebae, an important effect over both Madin–Darby canine kidney (MDCK) and Vero cell monolayers was observed (Figure 2). The main observed effect was a disruption of the monolayer by trophozoites, which were also attached to the plate in the spaces previously occupied by cells or between the attached cells. 

**Figure 3:**
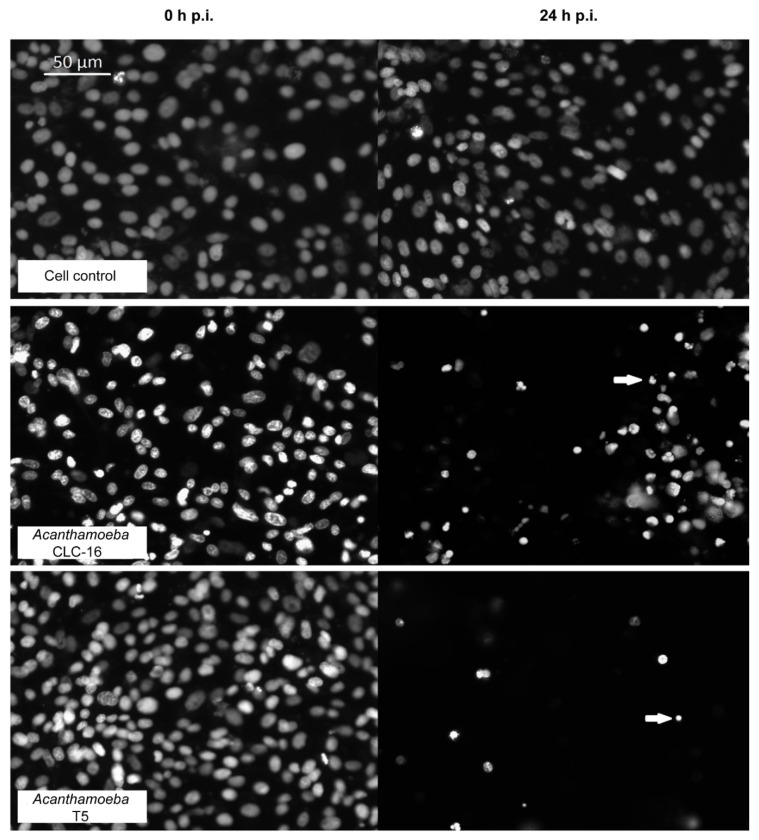
MDCK cell monolayers stained with the fluorescent stain Hoescht 33342 and incubated with *Acanthamoeba.* Images of the monolayer at 0 and 24 h postincubation with *Acanthamoeba* CLC-16 (genotype T3) (positive control of cytopathic effect) and *Acanthamoeba* genotype T5 are shown. Arrows indicate possible pyknotic cells.

An important decrease in the percentage of live cells after 8 h postinfection was observed for CLC-16 (positive control of cytopathic effect) and the *Acanthamoeba* T5 isolate using the Hoechst fluorescent stain (Table 1). After 24 h, only 14.3% of the cells incubated with the T5 isolate and 43% of the cells incubated with CLC-16 remained in the monolayer. Besides, some of the cells of the monolayer showed an alteration of the nucleus that resembled pyknotic cells (Figure 3). Monolayers without amoebae and monolayers incubated with a nonpathogenic *Acanthamoeba* genotype T4 (employed as a negative control of cytopathic effect) showed no alteration during the time of the experiment (Table 1). 

### 2.3. Secretion of Extracellular Vesicles by Acanthamoeba T5

#### 2.3.1. *Acanthamoeba* T5 Secrete Extracellular Vesicles in a Temperature-Dependent Way, with Different Sizes and Nanomechanical Properties 

A differential centrifugation protocol was employed to obtain EVs from *Acanthamoeba* T5 incubated at 28 °C and 37 °C. The resultant pellet was washed, the protein concentration was quantified and measurements were performed using dynamic light scattering (DLS) and atomic force microscopy (AFM). After the incubation of 4 × 10^6^ amoebae of the T5 genotype for 24 h in PYG culture medium for the release of EVs, no significant death of amoebae was detected (over 99% were viable) and 1.1 µg/µL of total protein of EVs were obtained.

Analyses by DLS revealed two populations of EVs with different sizes in *Acanthamoeba* T5 incubated at 28 °C (184.6 ± 50.80 nm and 50.29 ± 8.49 nm), while *Acanthamoeba T5* incubated at 37 °C secreted vesicles of 111.3 ± 19.8 nm (Figure S1). 

**Figure 4:**
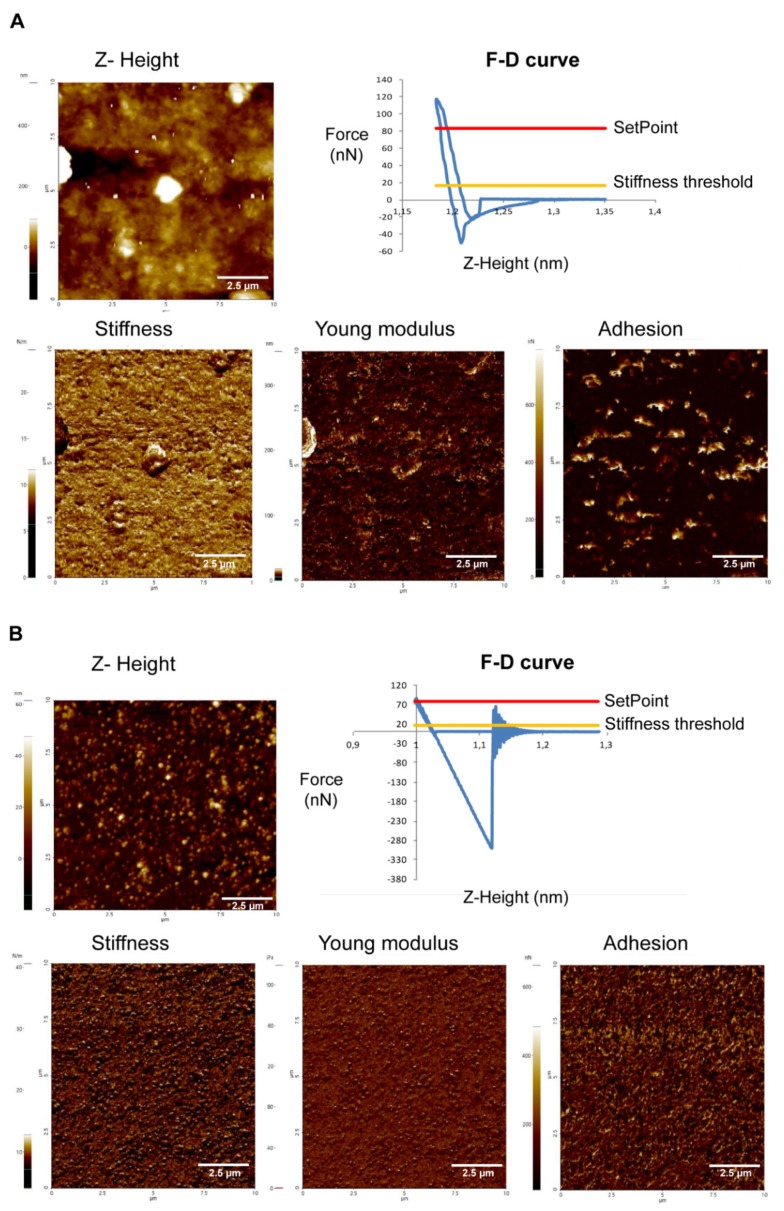
Atomic force microscopy Z-height images and nanomechanical maps showing stiffness, Young modulus and adhesion profile of the EVs of *Acanthamoeba* T5 incubated at 28 °C (**A**) and 37 °C (**B**) in PYG medium.

AFM was employed for both topography measurements and force spectroscopy of EVs of *Acanthamoeba* T5. Moreover, one of the objectives of this work was to evaluate the presence of possible differences in the mechanical properties between the EVs of this potentially pathogenic *Acanthamoeba* T5 incubated at 28 °C and 37 °C. Nanomechanical analyses demonstrated lower values of stiffness and Young modulus of the EVs of amoebae incubated at 37 °C when compared to isolated EVs of amoeba incubated at 28 °C (stiffness: 10.63 ± 0.6785 N/m in EVs of *Acanthamoeba* T5 at 37 °C and 16.75 ± 0.1175 N/m in EVs of *Acanthamoeba* T5 at 28 °C; Young modulus: 558.3 ± 27.25 GPa in EVs of *Acanthamoeba* T5 at 37 °C and 676.4 ± 72.69 GPa in EVs of *Acanthamoeba* T5 at 28 °C). However, adhesion was higher in EVs of *Acanthamoeba* T5 incubated at 37 °C (329.8 ± 21.75 nN) when compared to the EVs of the amoebae incubated at 28 °C (31.46 ± 11.06 nN) (Figure 4).

#### 2.3.2. EVs of *Acanthamoeba* T5 Incubated at 37 °C have Higher Proteolytic Activity than *Acanthamoeba* T5 Incubated at 28 °C and Their Cargo Includes Serine and Cysteine Proteases

**Figure 5:**
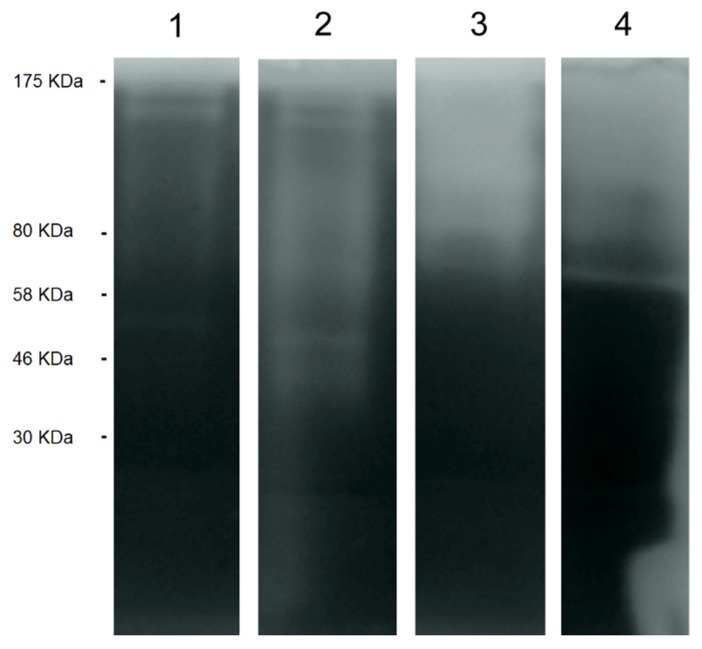
Protease zymogram of the extracellular vesicles (EVs) of the *Acanthamoeba* T5 isolate. For each lane, 8 µL of the sample at a concentration 1.1 µg/µL was loaded onto the gel. Lanes: 1) EVs of amoebae incubated at 28 °C, 2) EVs of amoebae incubated at 37 °C, 3) ACM of amoebae incubated at 28 °C and 4) ACM of amoebae incubated at 37 °C.

As in the case of the ACM, the presence of proteases in the EVs of *Acanthamoeba* T5 incubated at both temperatures (28 °C and 37 °C) was determined using zymography. Proteolytic activity of the EVs of amoebae incubated at 37 °C was higher than the activity of the EVs of the same isolate incubated at 28 °C and the ACM of amoebae incubated at both temperatures (Figure 5). Both PMSF and 2-iodoacetamide inhibited protease activity of the EVs and the conditioned media almost completely at both temperatures (results not shown), suggesting the presence of serine and cysteine proteases in the vesicles. None of the inhibitors acted over the ≈122 kDa band. 

## 3. Discussion

This work describes the pathogenic potential of an *Acanthamoeba* isolate from a water sample collected in a hospital located in the province of Chinandega, Nicaragua, and identified by sequencing as *Acanthamoeba* genotype T5 (accession number: MH82415) with 98% homology with the species *A. lenticulata* and thermotolerant at 37 °C. Thermotolerance is reported as an indirect factor of pathogenic potential and this characteristic is related to the ability of the amoebae to resist the body temperature of the host, which could be considered as an adaptive advantage when acting as a pathogen. On the contrary, the isolate was nonosmotolerant and this could limit its ability to resist high osmotic pressures, a situation that the amoebae could face when acting as a pathogen of the corneal epithelium [36]. 

In this study, the production of serine and cysteine proteases by the *Acanthamoeba* T5 isolate was proven. Early reports considered serine proteases as pathogenicity markers [37], with confirmed action of proteases in plasminogen activation, as well as collagen and fibronectin degradation [38]. In 2013, Omaña Molina et al. confirmed the role of proteases in tissue invasion, emphasising their participation in extracellular matrix digestion, but not in the direct process of cellular lysis [39]. A similar protease activity was demonstrated between the crude extract of trophozoites and the conditioned medium (especially in proteases between 48 and 80 kDa). In *Acanthamoeba,* proteases act upon plasminogen, collagen and fibronectin, and are considered important for invasion, degradation of connective tissue and alteration of the permeabilization of cells [40,41]. For *A. castellanii*, Ramírez et al. [42] suggested that proteases with weights between 50–250 kDa are cysteine proteases capable of producing the degradation of iron-binding proteins.

The virulence of the *Acanthamoeba* T5 isolate employed in this study was also demonstrated using the crystal violet and Hoechst dyes. At 20 and 24 h postincubation of the cells with amoebae, a higher cytopathic effect of the T5 isolate was observed when compared to the control strain (*Acanthamoeba* CLC-16) (*p* < 0.05). Using the CellProfiler software for the image analysis, it was possible to suggest the appearance of intracellular morphological changes such as pyknosis, although the most important difference was the percentages of live cells. A temperature-dependent secretion of extracellular vesicles by this *Acanthamoeba* T5 isolate was also evidenced in this study. As described in the results section, analyses by DLS revealed the predominance of two populations of EVs with different sizes in *Acanthamoeba* T5 incubated at 28 °C (184.6 ± 50.80 and 50.29 ± 8.49 nm), while *Acanthamoeba* T5 incubated at 37 °C secreted vesicles mostly of 111.3 ± 19.8 nm (Figure S1). Except for the population of 50.29 ± 8.49 nm, the size of the EVs obtained in both cases was similar to those reported in the two previous published studies to date, although these were performed using *Acanthamoeba* of the genotype T4. For example, in 2018, de Souza Gonçalves et al. [34] reported EVs of *Acanthamoeba castellanii* of 117.1 ± 73.3 nm in amoebae incubated in PYG medium and EVs of *Acanthamoeba castellanii* of 117.7 ± 55.8 nm in amoebae incubated in a glucose medium (under nutritional stress). More recently, Lin et al. [35] reported mean diameters of 166.7 nm in the EVs of the same strain of *Acanthamoeba.* Differences in size could be related to the methodology employed for the isolation and purification of the EVs and their composition.

It was demonstrated for the first time that the temperature of incubation of the amoebae is an essential factor for the secretion of EVs by *Acanthamoeba,* as we obtained differences in the protein quantity and size of the secreted EVs (Figure S1). In this sense, it is well known that temperature changes are stress inductors over cells (or amoebae, in this case) and this change forces them to perform a physiological adjustment that could influence the quantity, molecular composition of the surface and dynamics of the EVs released. In the case of parasites like *Leishmania* [27], *Trypanosoma cruzi* [23,24,25,43,44,45], *Toxoplasma gondii* [28,29] and *Plasmodium* [46], it has been proven that EVs carry diverse virulence factors and distinct types of small RNAs with possible roles in the host-parasite interaction [47]. Besides, in *Dictyostelium discoideum,* another free-living amoeba, the results of several studies raised the hypothesis of the participation of EVs in cell-to-cell communication [48,49]. Regarding *Acanthamoeba*, as mentioned before, there are two recent studies that demonstrated the production of EVs by trophozoytes of *A. castellanii* under homeostasis and nutritional stress and characterized their protein content [34,35]. The authors determined the lipid and protein content of these vesicles secreted by the amoebae incubated at 28 °C, temperature in which amoebae behave as free-living organisms. De Souza Gonçalves et al. [34] proposed that the EVs in FLA could participate in the regulation of their surrounding environment, including the amoebic population density and the regulation of symbionts´ gene expression. It was also suggested that EVs could play a role during the infection process of the amoebae to a host and that they could also induce the immune response in human cells [35].

In this study, by employing atomic force microscopy, it was possible to characterize and determine the nanomechanical properties of EVs of *Acanthamoeba* genotype T5. According to Sharma et al. [50], biomechanical properties of vesicles may play an important role in exocytosis and intercellular transport, but the quantification of these properties in living cells has been performed specially in studies that employ cancer cells [51,52]. For example, Xiao et al. [51] reported that human small airway epithelial cells (SAECs) were stiffer and more adhesive than cancerous A549 cells and, after the treatment of these cells with an anticancer drug for four hours, both biomechanical properties of A549 cells were increased. Whitehead et al. [52] also demonstrated that stiffness and adhesion of vesicles derived from malignant cancer cells were, on average, one order of magnitude lower when compared to nonmalignant cell exosomes and this could be related to metastasis of malignant cells. In our study, the results demonstrate the higher adhesion capacity and lesser stiffness in the EVs of *Acanthamoeba* incubated at 37 °C when compared to *Acanthamoeba* incubated at 28 °C. Adhesion is a characteristic considered as an important virulence factor in these organisms and is also a key step in the pathogenic process, precisely when the amoeba invade and parasitizes a host. These differences in adhesion could be associated with differences in the protein surface composition and the type of proteins present in the EVs of the amoebae incubated at 37 °C. Possible changes in the protein composition and nanomechanical properties of the EVs isolated at both temperatures could have implications in the parasite´s survival and its damaging potential in two radically different biological environments, so further experiments to characterize the proteomic content of EVs secreted at both temperatures are being carried out in our group.

Secreted EVs of *Acanthamoeba* T5 incubated at two different temperatures also showed differences in the protease content, as we observed a higher protease activity of the EVs obtained at 37 °C in the zymographic analyses using gelatin (Figure 5). The importance of the production of proteases in *Acanthamoeba* has been already discussed above. However, the higher capacity of the degradation of gelatin observed in the EVs of this T5 genotype incubated at 37 °C could suggest a role of EVs in the cytopathogenicity observed over the cell cultures and in their ability to degrade tissues and permeabilize cells. 

The finding of potentially pathogenic *Acanthamoeba* in water systems of a hospital like the one described in this study, no matter the genotype of the isolation, should alert the health authorities to consider these protozoan organisms as infectious agents, especially considering the state of vulnerability of the population that may be at risk in these sites. The presence of free-living amoebae (FLA) in water systems (including treated drinking water systems) and the risk that this could represent to the human health has been a subject of discussion for several years. In the Guidelines for Drinking-Water Quality published by the WHO [53], *Naegleria* and *Acanthamoeba* are considered as waterborne pathogens with a high impact in human health, and have low infective doses (1–10^2^ organisms). The Centers for Disease Control and Prevention (CDC) explain that these amoebae cannot be acquired by drinking contaminated water [54], and the WHO [53] raises the possibility that these organisms can be transmitted by water when it gets in contact with mucosae, skin wounds or the eye, especially in contact lens wearers. In a published review, Thomas et al. [55] indicated that, even when FLA are consistently detected in treated drinking water systems, more research is needed to evaluate the possible effect in human health, since few genotypes are potentially pathogenic and the infection route is not very simple.

Regarding *Acanthamoeba,* the role of domestic tap water as a potential source of amoebic keratitis has been reported, suggesting that older plumbing and poorly maintained water storage tanks may increase the risk of contamination with *Acanthamoeba* [56,57,58]. Recent studies report the isolation of *Acanthamoeba* T5 in tap water. Fallah et al. [59] published for the first time the presence of *Acanthamoeba* T3, T4, T11 and T5 in tap water sources from a touristic region in Iran. In another study about the distribution of *Acanthamoeba* genotypes in water samples from mineral water bottles in Porto Alegre, southern Brazil, the authors reported genotype T5 as the most common among the sequences analyzed [60]. Finally, Sente et al. (2016) [61] reported the isolation of *Acanthamoeba* in 36 domestic water sources (42.9%) in the Queen Elizabeth Protected Area, Uganda, including the genotypes T1, T2, T4, T5, T6 and T11. Even when *Acanthamoeba* genotype T5 is the second most frequent genotype isolated from environmental sources [1], there are few reports which correlate this genotype with a clinical case. The reasons for these few reports are unknown, since its pathogenic potential has been proven [62]. Ledee et al. [10] proposed that it could be possible that the majority of *Acanthamoeba* T5 isolates may not be pathogenic to humans, however, there are not enough publications to support this affirmation. Due to the severity of the clinical cases related to *Acanthamoeba* T5, Iovieno et al. [14] indicated that possibly the cases produced by atypical *Acanthamoeba* genotypes (like genotype T5) could be associated with a worse prognosis and resistance to therapy.

## 4. Materials and Methods

### 4.1. Axenic Culture of Acanthamoeba 

*Acanthamoeba* isolated from a water sample of a hospital was axenically grown in 25 cm^2^ cell culture flasks with 0.75% proteose peptone, 0.75% yeast extract and 1.5% glucose (PYG) medium. Amoebae were cultured and maintained as reported by Castro Artavia et al. in 2017 [63].

### 4.2. DNA Extraction and Isolate Genotyping

DNA from the axenic culture of *Acanthamoeba* was extracted using the QIAamp DNA Mini Kit (Qiagen, Hilden, Germany), following the manufacturer’s instructions. Approximately, 10^5^ amoebae were transferred to Eppendorf tubes and centrifuged at 7000 ×*g* for 10 min. The resulting pellet was employed for the extraction, with the elution step performed in a final volume of 70 µL. DNA was stored at −20 °C until further use.

Amplification of the Diagnostic Fragment 3 (DF3) region of the 18s rDNA gene was performed as previously described by Booton et al. [64] using the pair of primers JDP-1 and JDP-2 (Invitrogen, Waltham, MA). The thermal cycling conditions were the same as described by Retana Moreira et al. [18]. DNA from the strain *Acanthamoeba castellanii* Neff American Type Culture Collection (ATCC) 30010 and distilled water added to the reaction mix were used as positive and negative controls, respectively. 

PCR products were purified using the QIAquick PCR purification kit (Qiagen) and sequenced in both directions, in a MegaBACE 1000 automatic sequencer (Healthcare Biosciences, Madrid, Spain), as previously described [18]. The nucleotide sequence was analyzed and edited using the online tool DNA Baser v4.2.0 (Heracles Biosoft 2012).

### 4.3. Osmotolerance and Thermotolerance Assays

For the osmotolerance assays, amoebae were cultivated onto non-nutrient agar plates supplemented with *Escherichia coli* and 0.5 and 1 M mannitol. Approximately 1000 trophozoites were inoculated at the center of the agar plate and incubated at 30 °C for up to 72 h, with daily observation. Proliferation of *Acanthamoeba* sp. was determined by measuring the increase in diameter of the clearance zone in the bacterial lawn. For the thermotolerance assays, the same quantity of amoebae was inoculated onto non-nutrient agar plates supplemented with *E. coli*. Cultures were incubated at 37 °C and 40 °C. Proliferation of *Acanthamoeba* sp. was evaluated by inverted microscopic examination daily for up to 72 h. Each assay was performed in triplicate.

### 4.4. Preparation of Acanthamoeba Conditioned Medium (ACM) 

ACM was prepared by incubating confluent flasks of amoebae with 2 mL of RPMI-1640 medium (Sigma Aldrich Co., St. Louis, USA) for 24 h at 30 °C. After this time, cell-free conditioned medium was collected by centrifugation at 14,000 ×*g* for 5 min, filtered through a 0.22 μm pore filter (Minisart, Sartorius stedim Biotech GmbH, Goettingen, Germany) and stored at −80 °C.

### 4.5. Determination and Characterization of Protease Secretion by Zymography

Zymographic assays were performed as previously described by Herron et al. [65]. Briefly, protein extracts of trophozoites and ACM were subjected to electrophoresis on SDS-polyacrylamide gels (SDS-PAGE) containing gelatin (1 mg/mL) as described by Castro Artavia et al. in 2017 [60]. After the electrophoresis, the gels were washed twice in 1% Triton X-100 (w/v) for 30 min to remove the SDS and incubated overnight at 37 °C in a developing buffer (50 mM Tris-HCl, 10 mM CaCl_2_, pH 8.0), rinsed and then stained with Coomassie brilliant blue R-250 (Bio-Rad Laboratories, California, USA). Areas of gelatin digestion, which indicate protease activity, were seen as nonstained regions in the gel. To characterize the type of proteases produced, we followed the methodology described by Castro Artavia et al. [63], using phenylmethylsulfonyl fluoride (PMSF) 1 mM (Sigma Aldrich Co., St. Louis, USA) (inhibitor of serine proteases), 2-iodoacetamide 1 mM (Merck, Hohebrunn, Germany) (inhibitor of cysteine proteases) and EDTA 10 mM (Sigma Aldrich Co., St. Louis, USA) (inhibitor of metalloproteases). As EDTA is a reversible inhibitor, it was also included in the developing buffer. 

### 4.6. Evaluation of the in Vitro Effect of Acanthamoeba in Cell Cultures

Cell culture: Madin–Darby canine kidney (MDCK) epithelial cells (NBL2 ATCC CCL-34TM) and Vero cells (ATCC CCL-81) were grown in 75 cm^2^ cell culture flasks (Corning, Corning Incorporated, NY, USA) with RPMI 1640 medium (Gibco GranIsland, NY, USA) supplemented with penicillin (100 U/mL), streptomycin (100 pg/mL) and 10% fetal calf serum (Gibco, Gran Island, NY, USA). Flasks were maintained at 37 °C in a humidified 5% CO_2_ incubator. 

Cristal violet stain: Cell cultures were grown until confluence in 24-well plates (Costar Corning Cell Bind, USA) and the *Acanthamoeba* isolate (1 × 10^5^ amoebae per well) was added over the cell monolayers in serum-free medium. The incubation was performed during 24 h at 37 °C and the cytopathic effect was assessed macroscopically after staining the wells with crystal violet. *Acanthamoeba castellanii* Neff strain (American Type Culture Collection 30010) and *Acanthamoeba* T3 strain CLC-16 [66] were employed as positive controls of cytopathic effect. 

Fluorescent stain with Hoechst 33342: MDCK cells were cultured in a black polystyrene 96-well plate (Greiner Bio-One, Austria) with RPMI-1640 medium (Gibco GranIsland, NY, USA) supplemented with 10% fetal calf serum, glucose, antibiotics and without phenol red. Cells were incubated at 37 °C and 5% CO_2_ until reaching a confluency of approximately 80%. Then, the supernatant was carefully removed and the cells were stained with Hoechst 33342 (10 μg/mL) (Life Technologies, USA) in RPMI-1640 medium for 10 min. The cells were washed 3 times to remove the excess of dye and 100 µL of the amoebae culture (1 × 10^4^ amoebae/mL) was added to each well. The plates were incubated in the Cytation 3 cell imaging muti-mode reader (BioTek Instruments, Inc., USA) for 24 h at 37 °C and 5% CO_2._ An automatic image capture of 9 fields/well was performed every 4 h, for 24 h. For the quantitative analysis of the images, the software CellProfiler (Carpenter et al., 2006) was employed. *Acanthamoeba* T3 CLC-16 strain [66] was employed as the positive control, due to its known pathogenic capacity over different cell lines [67]. A monolayer of MDCK without amoebae was employed as the negative control. Nonpathogenic *Acanthamoeba* genotype T4 (GenBank accession number: KP677460), isolated, cultured and physiologically characterized in our laboratory was also employed in this assay as another negative control [19]. Each assay was performed in triplicate.

### 4.7. Isolation and Purification of the Extracellular Vesicles of Acanthamoeba T5

For the isolation and purification of the EVs of *Acanthamoeba* T5 trophozoites, we employed differential centrifugation, as described by Retana Moreira et al. [25], with some modifications. Briefly, 4 × 10^6^ amoebae were incubated for 24 h at 28 °C and 37 °C in PYG culture medium. After this time, the amoebae were centrifuged at 3500 ×*g* for 15 min and the supernatants were collected and centrifuged at 17,000 ×*g* for 15 min. The supernatants were filtered through 0.22 μm pore filters (Sartorius Minisart, USA) and ultracentrifuged again at 100,000 xg for 18 h to obtain the pellets with mostly exosomes. The pellets were washed 3 times in PBS, resuspended in 50 µL PBS and stored at 4 °C if employed immediately. All the steps were performed at 4 °C in an ultracentrifuge Avanti^®^ J-30I (Beckman Coulter, USA) with a JA-30.50 Ti rotor. The isolation procedure was evaluated by dynamic light scattering and atomic force microscopy, and the protein content of the EVs was quantified using the Micro-BCA protein assay kit (Thermo Fischer Scientific, USA) as described elsewhere [25]. Viability of the parasites after shedding of EVs was evaluated using the Trypan blue exclusion test. 

### 4.8. Dynamic Light Scattering of the EVs of Acanthamoeba T5

Samples of EVs of *Acanthamoeba* T5 incubated at 28 °C and 37 °C were diluted 1/100 in low-binding Eppendorf tubes with PBS and the particle movement was analysed using a Zetasizer nano ZN90 (Malvern Instruments, UK). Measurements were performed at 25 °C and data acquisition and information processing was performed using the Zetasizer Ver. 7.11 software.

### 4.9. Atomic Force Microscopy Imaging of the EVs of Acanthamoeba T5

EVs of *Acanthamoeba* incubated at both temperatures were diluted 1:10 in PBS and adsorbed onto freshly cleaved muscovite mica sheets for 10 min, the first choice for the AFM imaging of EVs [68]. After the adsorption process, mica sheets were rinsed with deionized water and dried under a gentle stream of nitrogen. Samples were analyzed using a Park AFM NX20 (Park Systems, Korea) at the Centro de Instrumentación Científica (CIC) of the University of Granada, employing the noncontact mode to construct the topographic images, using pyramidal-shaped silicon cantilevers with a spring constant of 40 N·m^−^ and a resonance frequency of 320 kHz. Images were typically acquired as 256 × 256 pixels at a scan rate of 0.5–0.7 Hz and analyzed using the XEI software (Park Systems, Korea). 

In order to detect possible differences in the nanomechanical properties of the EVs isolated at both temperatures, AFM was also employed using NSC-14 probes with a spring constant of 5 N/m and a resonance frequency of 160 kHz. Measurements were performed using the PinPoint^TM^ mode to acquire reproducible and reliable topography and elastic modules, adhesion and stiffness maps. Nanomechanical values were obtained from the force-distance curves (F-d curves) after the tip-surface contact. The values obtained were considered as relative values. 

### 4.10. Zymography of the EVs of Acanthamoeba T5

Zymographic assays were performed as previously described for the protein extracts of trophozoites and ACM, but employing the EVs obtained after the incubation of amoebae at 28 °C and 37 °C. Conditioned media collected at both temperatures were employed as controls of gelatin degradation. The type of proteases present in the EVs of *Acanthamoeba* T5 isolated at both temperatures was also characterized as previously described in Section 4.5. 

### 4.11. Statistical Analysis

Means and standard deviations of the percentage of live cells observed every 4 h were calculated for each isolate. A one-factor ANOVA was performed to detect significant differences between the isolates, after proving the normal distribution and homoscedasticity of data. In order to determine which of the isolates had statistically different means, multiple comparisons post hoc using the Tukey test were performed. Values with *p* < 0.05 were considered statistically significant. 

## Figures and Tables

**Table 1:** Percentage of MDCK cells in the monolayer. Hoechst stain of the nucleus of live cells incubated with different *Acanthamoeba* genotypes for 24 h.

Time p.i * (h)	*Acanthamoeba* CLC-16 (Genotype T3)	*Acanthamoeba* (Non-Pathogenic Genotype T4)	*Acanthamoeba* Genotype T5	Cell Control
0	100.0	100.0	100.0	100.0
4	98.7 (± 3.7)	109.9 (± 2.6)	101.8 (± 4.9)	104.3 (± 1.5)
8	67.4 (± 7.4)	108.5 (± 2.3)	88.9 (± 6.2)	107.4 (± 1.5)
12	45.2 (± 6.3)	109.2 (± 3.5)	68.0 (± 1.6)	105.9 (± 1.8)
16	38.7 (± 4.7)	107.1 (± 5.5)	42.3 (± 5.5)	105.5 (± 7.0)
20	41.3 (± 3.7)	103.2 (± 5.0)	22.4 (± 2.3)	106.4 (± 5.0)
24	43.0 (± 3.2)	102.1 (± 7.5)	14.3 (± 2.7)	103.9 (± 2.8)

* Postincubation.

## Supplementary Materials

The following are available online at https://www.mdpi.com/2076-0817/9/2/144/s1, Figure S1: Isolation of EVs of *Acanthamoeba* T5 using differential centrifugation: (A) *Acanthamoeba* T5 in PYG medium, (B) final pellet obtained after the centrifugation of the *Acanthamoeba* T5 supernatant at 100,000 xg for 18 h, (C) AFM topography images of EVs of *Acanthamoeba* T5 incubated at 28 °C, (D) AFM topography images of EVs of *Acanthamoeba* T5 incubated at 37 °C, (E) DLS of EVs of *Acanthamoeba* T5 incubated at 28 °C and (F) DLS of EVs of *Acanthamoeba* T5 incubated at 37 °C. The vesicle population size of 569.5 ± 116.2 nm could correspond to aggregates of EVs.
