# Supplementary material for: Isolation of Acanthamoeba T5 from Water: Characterization of Its Pathogenic Potential, Including the Production of Extracellular Vesicles
Source: Pathogens. 2020 Feb 21;9(2):144. doi: 10.3390/pathogens9020144 (PMC7168589; doi:10.3390/pathogens9020144)
Supplement: Supplementary file 1 [file pathogens-09-00144-s001.pdf]

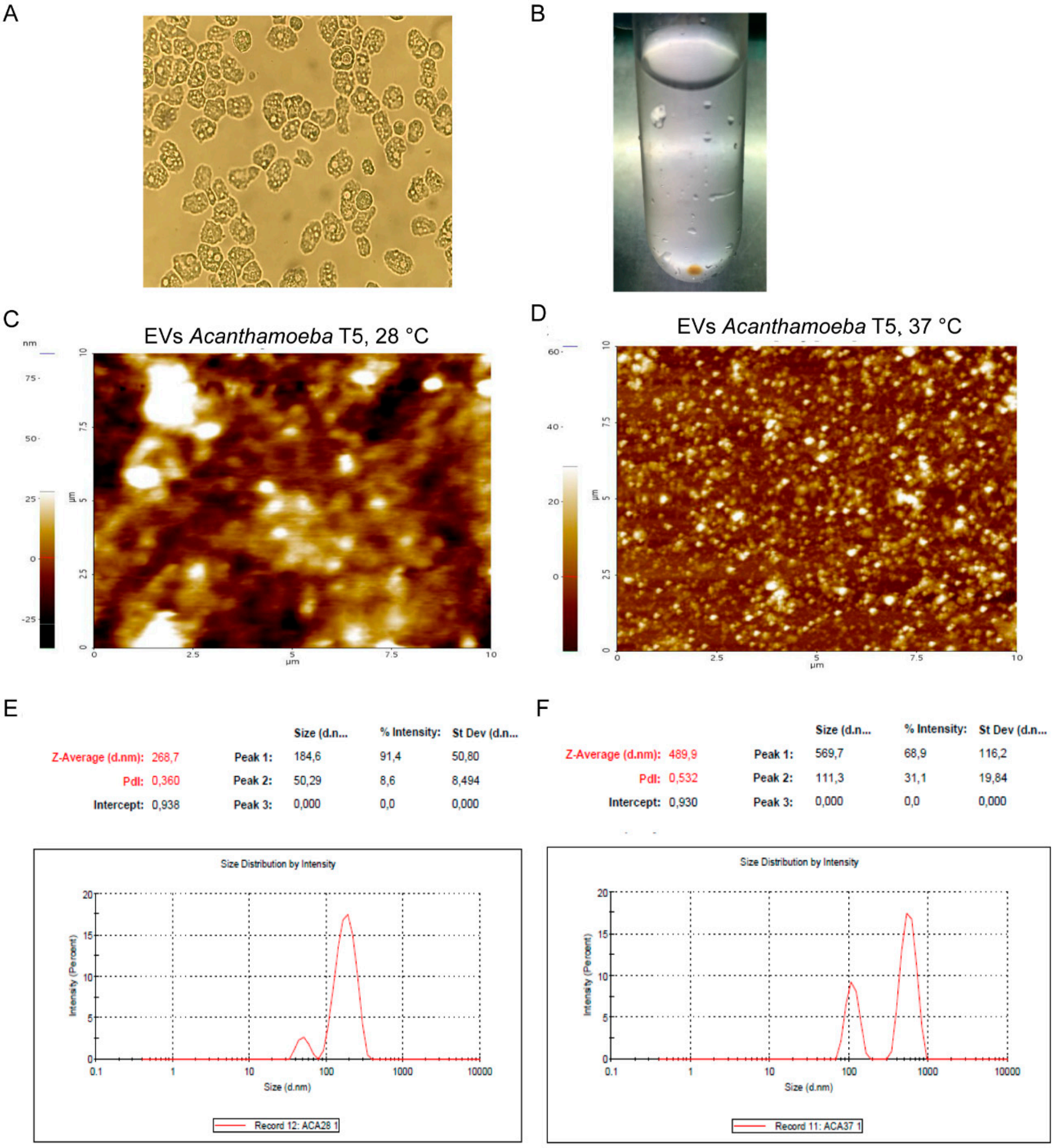

Figure S1. Isolation of EVs of *Acanthamoeba* T5 using differential centrifugation: A) *Acanthamoeba* T5 in PYG medium, B) final pellet obtained after the centrifugation of the *Acanthamoeba* T5 supernatant at 100,000 xg for 18 h, C) AFM topography images of EVs of *Acanthamoeba* T5 incubated at 28 °C, D) AFM topography images of EVs of *Acanthamoeba* T5 incubated at 37 °C, E) DLS of EVs of *Acanthamoeba* T5 incubated at 28 °C and F) DLS of EVs of *Acanthamoeba* T5 incubated at 37 °C. The vesicle population size of 569.5 ± 116.2 nm could correspond to aggregates of EVs.
